# Root-Canal Fillings

**Published:** 1903-07-15

**Authors:** John I. Hart


THE
DENTAL REGISTER.

Vol. LVII. July 15, 1903. No. 7.

### ROOT-CANAL FILLINGS.

BY JOHN I. HART, D.D.S.

The properties that should be possessed by a canal filling are as follows:

(1) It should be capable of completely filling the canal and sealing the apex.

(2) It should possess antiseptic properties.

(3) It should be capable of easy insertion.

(4) It should be durable.

(5) It should be pliable and moldable.

(6) It should neither expand nor contract.

(7) It should not discolor the teeth.

(8) It should be impermeable to fluids.

(9) It should be easily removable from the canal.

As you are well aware, we have many root-canal fillings, all of which possess some of the requisites mentioned. In my estimation none of those which are recognized as standard root-canal fillings comply with all these requirements. The one most extensively employed is the zinc oxychlorid, but it falls short of the last mentioned requirement. Theoretically speaking, it is not a difficult matter to remove from a root-canal a zinc oxychlorid filling, but in nearly all instances when it is necessary to remove such a filling, it is because of pericemental irritation which renders the tooth particularly sore to the touch, and the last third of the filling is exceedingly difficult to remove and a source of irritation to patient and operator.

In my practice, gutta-percha in root-canals is only of use in sealing the apex of a root which has been enlarged and is to receive a dowel or a post to support a crown; having enlarged the canal, I know when I place the gutta-percha that it is carried to the point which I desire; and in the event of the post loosening, the crown can be reset without fear that infection of the apical space may have occurred; or, in roots which possess unusally large apical openings, I feel safer in filling with gutta-percha, but in a normal root I know of no canal filling which comes closer to our demands than iodoform compound and a tin point.

Knowing the prejudice that exists against iodoform on account of its disagreeable odor, I will pass around for your observation the iodoform compound, in which, owing to the essential oils, the odor is not only decreased, but I think you will not find it perceptible. For those who deem iodoform too inert for this purpose, aristol or hydronaphthol might be substituted. Tin points, which I here show you, are made so that they can be inserted in the finest canal or in larger canals.

Lead points of a similar shape are furnished, but in my hands are not so satisfactory as the tin, being a trifle less stiff than are the tin points. The earliest root-canal fillings were made with gold foil and shredded tin foil, as indicated by Dr. Henry H. Burchard in his article on the treatment and filling of root-canals in the "American Text-Book of Operative Dentistry," as well as gold, copper, and lead points, and wood points dipped in creosote.

In that article I find no mention of tin points, and in conversation with my professional friends I have found that they are not extensively used; in fact, when I desired to get my last supply from the dental depot I was informed that they had given up their manufacture. At my solicitation they accepted an order for a quantity to be made up as a special order.

In Dr. Burchard's article he suggests that "the readily oxidizable metals have not found favor, owing to the possibility of dentinal staining following their employment." I have not found, however, that the tin points, used as I shall suggest, present this difficulty. When the canal is ready for for filling I measure its length and cut off a section of tin point from some part of the point corresponding to the circumference of the canal, cutting the point somewhat longer than the canal, so that when it is inserted it can be turned over into the pulp-chamber. Then I take a small quantity of the iodoform compound on a smooth broach and pump it into the canal to about half its capacity, taking care that it is carried to the apical end; if any excess be spread on the pulp-chamber wall it is readily removed with spunk. The tin point is passed quickly through a flame, for the purpose not of heating but of sterilizing it, and is then placed in the root-canal, turning over the excess of tin. An oxyphosphate of zinc is placed over the root-canal or canals, and our tooth is ready for its filling.

If there is never any subsequent trouble, we have a canal-filling which, in my estimation, answers all the requirements and which for several years it has been almost my exclusive practice to use. If unfortunately it becomes necessary to open such a tooth for treatment, any time after we reach the pulp-chamber, we can withdraw the tin point and we have a canal ready for treatment without subjecting our patients to the annoyance of the tedious removal of another filling material.

### DISCUSSION.

Dr. Hatch: I would like to know just what this iodoform compound is, of which Dr. Hart speaks.

Dr. Hart: Iodoform, zinc oxid, and oil of cloves.

Dr. Hatch: Do you make it yourself?

Dr. Hart: No; it is prepared in Philadelphia by Dr. Foulkes.

Dr. M. L. Rhein: I do not think the profession has found as yet the ideal root-filling. My own opinion is that of all the fillings we have at present, chloro-percha followed up with gutta-percha comes nearer to what we are aiming at than anything the profession has at its disposal. Very little attention has been given to the sterilizing of gutta-percha and chloro-percha before using, and I have failed to hear any detail given to that point; but it is a very easy thing to sterilize gutta-percha with formaldehyde before using it, to overcome any possible infection that may have clung to the material. Personally, I think there ought to be no occasion for the removal of a root-filling.

Of course, this brings us back to the ideal condition spoken of in a general way by Dr. Perry in his beautiful paper—a condition of affairs that never should necessitate the removal of a root-filling; and I do not believe that a practitioner who endeavors to reach, as far as possible, that point should seriously entertain the question of the removal of that filling, as far as any of the trouble related by Dr. Hart is concerned.

There is a possibility in crown-work of the necessity of going up into a root, but there we are met with the staple of the crown, not the root-filling. It brings up a different condition of affairs. I simply cite that point as bearing upon the paper that preceded it. It eliminates in my own mind one of the conditions that the essayist laid down as a necessary qualification for root-filling.

Dr. Hatch: Dr. Hanning, speaking of the Soderberg mixture and Dr. Hart's mixture, makes me think we should give a word of caution to the younger men who read this discussion, about placing too much dependence upon the antiseptic properties of permanent root-fillings. I take it that there is no antiseptic which will be permanently effective, and the nearer we approach to filling the root in a mechanically perfect way the better off we are. The principal reason why I would use an antiseptic would be the reason Dr. Rhein spoke of—the prevention of any infection then and there. Antiseptics do not stay permanently in teeth, no matter what we put in.

Dr. M. L. Rhein: I agree so thoroughly with the remarks just made that I want to correct a possible error in what I said. If I said chloro-percha, in regard to formaldehyde, I did not mean that; I referred more to the gutta-percha points. It has been my custom for some time to have those lying in a solution of formaldehyde, and immediately after removing them from that solution to put them into the canal, in order to obviate any possible infection that might cling to the point. Dr. Hatch has struck the keynote of root-sterilization. I would like to echo the strongest affirmative response to that. Our only means of avoiding infection through instrumentation, in the preparation of the root-canal, or by chemical means, is by having a root so free from organic matter that infection from anything remaining in the canal becomes an impossibility. If that be accomplished and every precaution be taken, root-canal trouble becomes almost an impossibility when the canals are sealed.

Dr. George Evans: There is this about the subject of root-fillings that I do not understand: how a whole profession seems so blind on certain points regarding the infection of root-canals. It seems as simple to me as anything can be, and I have followed it out for years. I have practiced this method for some time and can set the time exactly. I moved from Thirty-fourth street to Thirty-ninth street ten years ago next month; and in that time not one single tooth from which I removed the pulp has come back abscessed. It is done on a principle as simple as A, B, C, and I cannot see why it has not been understood. I promised the Institute of Stomatology a paper on that subject three years ago, and I never gave it because I did not get time to write it. I made that statement before some of the members, and they asked me to bring it before the society.

Members get up and state that they fill root-canals with cotton, gutta-percha, wood, and other things, and they have varied success. What does root-canal filling depend on? Does it depend on closure of the apical foramen? There is where they all break down—they talk about the apical foramen. How many teeth become infected through the apical foramen? Not one in a thousand. I have a case now that I am watching. The patient fell on the ice a short time ago, and the central was pushed far in. I put her in the chair and with my fingers forced it up. Tears came to her eyes but it brought the tooth up into position. It would have been a serious thing if the alveolar process had not united, and I watched it; it has united and grown fast. The last time she came in, about a week ago, to my annoyance I found the pulp had died and the tooth was slightly discolored. I am going to leave it there and see if it abscesses. So far it has not.

The whole secret is not in closing the apical foramen, as dwelt upon so carefully by Dr. Hart; it is in the closure of the canal! How does the pulp-canal become infected? Ninety-nine times out of a hundred, from the oral cavity. Fill a root-canal with anything you wish in an extracted tooth; put it on the shelf, leave it there for a time, open it, and there is no odor there; but if the tooth were in the mouth it would be different.

I was conversing with Dr. Perry some time ago, and either he or the gentleman associated with him said that he practically fills the end of a root-canal, closing the foramen with gutta-percha and filling up the rest of the canal with oxychlorid. He probably has great success with it. Why? Because oxychlorid is an antiseptic filling in itself. It is one of the densest cements we have, and it closes and hermetically fills up that orifice. Here is the whole secret: hermetically seal up the orifice of your cavity; keep out infection from the oral cavity through the secretions of the mouth. I do not care even if your filling is loose, if you have the orifice and root-canal hermetically sealed, no infection will take place; but if you fill it with gutta-percha, for instance, no matter how much you sterilize it, filling your root-canals entirely and then placing in an amalgam filling, the patient, ten chances to one, will come back with trouble there. Why? Because all amalgam shrinks, and so the cavity becomes infected around that amalgam. It gradually works up around the gutta-percha, which is never an air-tight filling, and works up around the canal, and infection takes place.

All my success in that line depends on this. The removal of the contents of the pulp-canal is necessary; then the filling of that so that the orifice is hermetically sealed. Some time ago a patient of a most respected friend of ours in New Jersey, a splendid operator, came into my hands, and for some reason I had to remove a gold filling. It was a splendid filling, adhesive gold, packed tight to every part of that cavity and up the root-canal to a slight extent; and, to my astonishment, after getting out the filling, I found the whole root-canal filled with a wad of cotton. I carefully removed it, passed it under my nose, and there was not a particle of odor. How did it stay there all that time? The apical foramen was open, because he had treated an abscess there. How did that occur? He was a splendid operator and he had put in an air-tight, water-tight filling and it had preserved the cotton intact all those years. There was another self-evident proof that I am right. I will not speak about diseased teeth that come into my hands, but where the root-canals have been prepared by me from the beginning; that has been my record, and I did it entirely upon this principle, as I have told you. Infection does not take place from the apex, but from the oral cavity. The orifice of the canal should be hermetically sealed and zinc oxychlorid is the thing to do it with, filling a little of the cavity with it under your filling. That is the whole secret.

Dr. Rhein: I have enjoyed listening to Dr. Evan's earnest remarks. I do not question in the least the results he has accomplished, and I trust he will pardon my simply disagreeing with him as to why he gets those results. My impression is he gets the results for the very reason he spoke of before—because his work in cleansing out the root-canal is absolutely thorough and he has left none of the organic matter in the canalculi of the root. I must differ with him as to the remark that it is impossible for root-canals to become infected except through the entrance into the canal. I do not doubt that such infection can take place, but I really believe those are the exceptions. It is very true that the infection does not all come from what he terms the apical foramen. There are very few roots that have any foramina inside the roots; the infection can come from those which are almost microscopic in character, and it can come from the peridental membrane; but the point I wish to emphasize is that such infection cannot proceed in a canal that has been thoroughly cleansed of organic matter. That is the reason of Dr. Evans' success, and it is the reason of the success of all of us when we do thorough work. I simply want to draw his attention to this little pathologic point, on which I take the liberty of disagreeing with him, in stating that there are a great many entrances by which infection in root-canals can be effected, and that the most common is through some circulatory form beyond the gingival margin. I take issue on that point.

Dr. Evans: I said that for ten years I had followed that method, and for the first few of those ten years I did not use sulphuric acid as I use it now, and I did not go to the apex of my canals in many cases, especially some of the canals in molars; in many of them I left them right there, using a saturated solution of aristol in oil of cloves. As to filling tubuli, I never filled any in my life; but I have played the mischief with them with the essential oils and aristol, and, in the beginning, iodoform; but what is the use of having your place filled with the odor of iodoform when you have in aristol all the properties you desire? What is the great essential in iodoform, coming down to the science and pathology of it? It is the nascent iodin that is liberated that comes in contact with organic matter. Iodoform enters into combination with hydrogen sulphid and forms iodic compounds that are liberated. With aristol you get nascent iodin liberated as rapidly in the tooth-canal as you want it.

Put it in solution with oil of cloves, on one of your probes. Leave it there for a while. You will find iron iodid has already to a certain extent been formed there and you can get it without using iodoform in the manner described. I used to use it, but I have not needed it for years. The perfect sterilization and mummification, with essential oils, of any remnants of organic matter left in root-canals should be done first; then fill the root-canal as well as you can, and hermetically fill the roots. How a canal gets infected in that way, I do not know; I do not take stock in infection through the pericementum. I cannot say I have seen a tooth affected in that way, with all due respect to Dr. Rhein.

DR. HANNING: I would not like to go on record as advocating the use of this Soderberg mixture, simply on account of its antiseptic qualities. The manufacturers of it said it would mummify the pulp. I get my broaches up as far as I can, and then put in a good, tight filling; I do not know whether my success is due to that or not. The very first time I used it I made a failure. The tooth was already affected. A druggist who was in my chair said, "suppose you try it on my tooth." I used some formaldehyde, and the tooth was filled up; that evening he went to one of his friends in his neighborhood and had the tooth extracted. It began to swell.

DR. DAILEY: There seems to be a divergence of opinion. I heard a statement that eucalyptus should be used. If the gentleman will try eucalyptol, the absolute drug, and also bergamot absolute, and the cinnamon, he will do away with the irritating quality in the commercial oil. The use of essential oil and its antiseptic value is greater than anything I know of.

DR. PERRY: I think this paper is a model for conciseness and correctness. It hits the nail on the head. It is not a big head either, and you must strike it squarely.

Mr. President, I wonder how long ago it is that you mentioned, in a paper called "Offerings," the little round bibulous paper points for drying root-canals?

The President: At least twenty-five years.

Dr. Perry: Probably a year after that, I commenced the use of the gutta-percha points. They were suggested by your paper. I thought if the little papers would dry it well, the little points would fill it well, and with a little instrument, held in the lamp, these were rolled out; after a number of years I presented them before this society—red gutta-percha points. I never had the credit of it, but that does not matter. I used them for a number of years, but I found after awhile that, although they seemed to fit well and the root-canals seemed to be dry enough around them, they almost invariably came out with a bad odor. Feeling sure that there was germ action going on wherever there was a bad odor, I went back to filling with zinc oxychlorid, because I had seen for so many years that this material used by all kinds of operators—good, bad and indifferent—seemed to do well. It was only a natural conclusion to believe that it was the antiseptic property in it that was that saving quality. There was danger in using it in cavities that were large at the apex, so there seemed nothing to do but to close the apex with little points of gutta-percha, and I used to sometimes even measure the size of the opening. I always measured the length with little barbs and I sometimes measured the opening with a little gutta-percha point warmed, and then perhaps filled it up with that same little point by pushing it gently.

Dr. Atkinson used to say, "Always fill the tooth solidly and firmly, and, if it need any treatment, help it to break out or let it break out and fill it from the outside." I think you will find a repetition of that statement for a number of years. I never agreed with that. I am a coward and timid; I don't want to have a patient suffer very badly; I would not like to be in that position myself. I have always filled them with a little undercurrent of fear that there might come a time when the filling would have to come out, and I must agree with Dr. Hart in his desire to use something which will come out easily if it be necessary to remove it. I must agree also with Dr. Rhein that the ideal filling has not yet been found, and I must agree with Dr. Evans in the main that infection often comes from the outside; but I believe in closing both ends.

Perhaps some of the younger men may think I am careless, but I do not care so much what I fill the canal with, so long as the apex is closed, because I expect to fill the other end accurately with my filling. I have seen the ends filled with cotton, without a particle of odor; and if there be no odor I have no fear. Dr. Evans said he has no trouble with teeth that he has devitalized. We have no trouble with the teeth *we* devitalize; that is a horse of another color; but it is the diseased teeth that bother us—those that have been dead for some time. There comes the opportunity for search for a more perfect filling than we have.

I gave up the gutta-percha points because of their bad odor, due to their absorption of secretions. Take them out after they have been in the tooth for a number of years, hold them over a lamp or put them under your nostril and you will find a little odor from them every time. Gutta-percha swells, and it is natural that it absorbs. I fell back upon what the older members had used for a long time so empirically, but so successfully, the zinc oxychlorid; it had the quality which Dr. Atkinson used to favor, but which I did not—that you could not take it out. It is a hard filling to get out, and a hard filling to get in, because the air is sure to get in and you have to work very accurately to fill those little ends with zinc oxychlorid; so after being dissatisfied quite a number of years in trying to fill with this material carried with cotton or silk into the apex, I took a hint from Dr. William Morrison, who used to fill the canals with gold wires, three or four of them—making sometimes the best filling and sometimes the very worst I have seen—reasoning that in those little hair-like canals a bit of silk or cotton twisted on a fine jeweler's broach would make a perfect filling if you could leave it up there. But, as you could not leave it, why not take a little fine gold wire, roughening it a little and twisting on it a few fibers of silk, and there you have a duplicate almost of the instrument with which you clean the root; then take the zinc oxychlorid, mixed very thin, first having swabbed the canal up to the apex, in order to be sure you have it wet, with the very thin oxychlorid; and then, with the fine gold point with the zinc oxychlorid wrapped around it and carried up to the very end, you know you have it filled; and I think it is one of the best things ever used by the profession—I came to that conclusion because of the observation of those old empirical cases—leaving a little portion extending into the pulp-chamber, so you have enough to get hold of, if you have to remove it. I think to-day there is no way of filling those very minute cavities on the buccal sides of the upper molars and in the bifurcations of the lower molars, as well as with a little gold wire filed down and fitted as accurately as possible, put in as I have said.

This filling of Dr. Hart comes the nearest to that idea, and it may be better in that it can be more easily removed than even the gold wire with the oxychlorid. What I should fear is that it is not quite stiff enough on the point sometimes to carry it where you wish. It might double up when the gold wire would not. Do not use soft gold; 14-k or 15-k. tempered gold is better—one which is stiff and springy and which will carry it up to the end. Sometimes I fill an upper molar with those little gold wires on the buccal side, and sometimes use the red gutta-percha points in the palatal root. That I have done sometimes in the last few years.

When it comes to the filling of the pulp-chamber, I do not know what to fill it with to-day. Dr. Jack has written me twice within a year on the subject of two split teeth, one of which was under my observation and another which he told me of, which he thinks were split by the expansion of oxychlorid in the pulp-chamber. I never saw a case which I could be sure was caused by the expansion of the zinc oxychlorid, except this one which came into my hands. It was a good, sound tooth, split right open. Within three or four weeks Dr. Jack has written me of another tooth split in the same way, and he said he was about to abandon the use of oxychlorid in the pulp-canals.

There is one bad feature even with the gold wire: While you may be very sure of getting the fit accurately near the apex of the root, you are liable to have a space around the end of the root as it approaches the pulp-chamber; and if you have not enough material to flow back and fill that up, you have a nasty place which is hard to fill. I think that perhaps Dr. Hart's method is better, because you can fill it well before you put the tin wire there and you can take the tin out any time you wish, which I think is essential. I do not think an absolutely-perfect filling has been found, and that is why you find so many people using different things. I have thought sometimes one of the best fillings we could have, if it could be made, would be such as Dr. Maynard used to make, which could not be removed, but which gave perfection of fit. I have several times within the last twenty years opened teeth which he filled with gold and observed how perfect they were. I have seen the instruments with which he put in the gold—each little piece packed in solidly, giving assurance by the sense of feeling that they were perfectly filled. There were no cesspools there and no chance of weeping from the end of the root. That is where I think Dr. Evans is mistaken; I think fluid can weep into it. I think it can run down and moisture can get in there when the filling is absolutely tight. So it is best to fill both ends absolutely tight, and then in the middle it is not so important.

Dr. Evans: I spoke in regard to the infection. I invariably close the apex of the canal most carefully with a little point of gutta-percha, measuring it carefully. I cork it, as I say, and fill one-third, sometimes one-half, with gutta-percha, and then fill the remainder with oxychlorid. I have found nothing in recent years which surpasses or even equals Ash's rock cement. I carry it up there generally with a few fibers of cotton.

Dr. Hart: I stated what I liked to do after the canal was prepared, and I purposely did not enter into the treatment of that canal, because I think we are all of one mind that it must be in an aseptic condition, even if we are not all agreed just as to what we will put in. Concerning infection from the apical space, I have seen a central incisor that had received a blow but which had no cavity. As a result the pulp died and an abscess followed; and how infection took place except through the circulation I cannot say. If Dr. Evans be correct it would become unnecessary to do any more than seal the pulpal end of root-canals with oxychlorid, put in a good filling, and we would have no trouble.

As to any odor connected with this material, if you can find that that is a disagreeable thing to use, I will concede that it is; at present I do not. I have tried the mixture of aristol and the essential oils. It is not as thin, and it becomes somewhat sticky and gummy—there is the difference between working gum and a thin, vaselin-like mixture.

It would be inexcusable for any one of us to have any septic irritation after devitalizing and surgically removing a pulp, and I am not wasting your time or mine considering such cases. I am speaking of cases where a pulp has become purulent after exposure; treating that tooth as well as we can with chemical sterilizers or by heat, we get it in a condition where there is no pericemental irritation, and it is aseptic and ready to fill. I want to put a filling in there that I can remove. I am not arguing with any of you as to how we can treat roots after we destroy the pulps, or after using some local anesthetic removing those pulps, but as to how thoroughly we can sterilize the dental tubuli. Once we treat a root that has been the seat of peridental inflammation, we know that one inflammation predisposes to another, and it may end by suppuration. Until our knowledge of bacteriology is changed, I want a root-canal filling that if trouble arises I can remove.—*Cosmos.*

